# Risk stratification by residual enzyme activity after newborn screening for medium-chain acyl-CoA dehyrogenase deficiency: data from a cohort study

**DOI:** 10.1186/1750-1172-7-30

**Published:** 2012-05-25

**Authors:** Catharina M L Touw, G Peter A Smit, Maaike de Vries, Johannis B C de Klerk, Annet M Bosch, Gepke Visser, Margot F Mulder, M Estela Rubio-Gozalbo, Bert Elvers, Klary E Niezen-Koning, Ronald J A Wanders, Hans R Waterham, Dirk-Jan Reijngoud, Terry G J Derks

**Affiliations:** 1 Section of Metabolic Diseases, Beatrix Children’s Hospital, University of Groningen, University Medical Centre of Groningen, PO Box 30 001, CA84, 9700 RB, Groningen, The Netherlands; 2 Laboratory of Metabolic Diseases, Department of Laboratory Medicine, University of Groningen, University Medical Centre of Groningen, PO Box 30 001, CA84, 9700 RB, Groningen, The Netherlands; 3 Center for Liver, Digestive and Metabolic Diseases, University of Groningen, University Medical Centre of Groningen, PO Box 30 001, CA84, 9700 RB, Groningen, The Netherlands; 4 Institiute for Genetic and Metabolic Disease, Department of Paediatrics, Radboud University Medical Centre Nijmegen, Nijmegen, The Netherlands; 5 Centre for Lysosomal and Metabolic Diseases, Department of Paediatrics, Erasmus Medical Centre, Rotterdam, The Netherlands; 6 Department of Metabolic Diseases, Academic Medical Centre, University of Amsterdam, Amsterdam, The Netherlands; 7 Department of Metabolic and Endocrine Diseases, Wilhelmina Children’s Hospital, University Medical Centre Utrecht, Utrecht, The Netherlands; 8 Section of Metabolic Diseases, Department of Paediatrics, VU University Medical Centre, Amsterdam, The Netherlands; 9 Department of Pediatrics and Laboratory of Genetic-Metabolic Diseases, Maastricht University Medical Centre, Maastricht, The Netherlands; 10 Laboratory for Infectious Diseases and Perinatal Screening, National institute for Public Health and the Environment (RIVM), Bilthoven, The Netherlands; 11 Laboratory Genetic Metabolic Diseases, Academic Medical Centre, University of Amsterdam, Amsterdam, The Netherlands

**Keywords:** Population newborn screening, Enzyme, Genotype, Prevalence

## Abstract

****Background**:**

Since the introduction of medium-chain acyl coenzyme A dehydrogenase (MCAD) deficiency in population newborn bloodspot screening (NBS) programs, subjects have been identified with variant *ACADM* (gene encoding MCAD enzyme) genotypes that have never been identified in clinically ascertained patients. It could be hypothesised that residual MCAD enzyme activity can contribute in risk stratification of subjects with variant *ACADM* genotypes.

****Methods**:**

We performed a retrospective cohort study of all patients identified upon population NBS for MCAD deficiency in the Netherlands between 2007–2010. Clinical, molecular, and enzymatic data were integrated.

****Results**:**

Eighty-four patients from 76 families were identified. Twenty-two percent of the subjects had a variant *ACADM* genotype. In patients with classical *ACADM* genotypes, residual MCAD enzyme activity was significantly lower (median 0%, range 0-8%) when compared to subjects with variant *ACADM* genotypes (range 0-63%; 4 cases with 0%, remainder 20-63%). Patients with (fatal) neonatal presentations before diagnosis displayed residual MCAD enzyme activities <1%. After diagnosis and initiation of treatment, residual MCAD enzyme activities <10% were associated with an increased risk of hypoglycaemia and carnitine supplementation. The prevalence of MCAD deficiency upon screening was 1/8,750 (95% CI 1/7,210–1/11,130).

****Conclusions**:**

Determination of residual MCAD enzyme activity improves our understanding of variant *ACADM* genotypes and may contribute to risk stratification. Subjects with variant *ACADM* genotypes and residual MCAD enzyme activities <10% should be considered to have the same risks as patients with classical *ACADM* genotypes. Parental instructions and an emergency regimen will remain principles of the treatment in any type of MCAD deficiency, as the effect of intercurrent illness on residual MCAD enzyme activity remains uncertain. There are, however, arguments in favour of abandoning the general advice to avoid prolonged fasting in subjects with variant *ACADM* genotypes and >10% residual MCAD enzyme activity.

## Background

Medium-chain acyl-Coenzyme A dehydrogenase (MCAD [E.C.1.3.99.3]) deficiency (OMIM 201450) is the most common inherited disorder of mitochondrial fatty acid oxidation. The MCAD enzyme is responsible for the first step in the mitochondrial β-oxidation of CoA esters of medium-chain length fatty acids [1].

Before the introduction of population newborn bloodspot screening (NBS) for MCAD deficiency, patients presented clinically during periods of catabolic stress, precipitating acute symptoms [2,3]. Some patients developed seizures, coma or even presented with sudden death, associated with hypoketotic hypoglycaemia. However, asymptomatic family members have also been recognized with the same disease-causing genotype [4]. Worldwide, approximately 80% of clinically presenting patients were homozygous for the c.985A>G missense mutation in the *ACADM* gene enco-ding the MCAD enzyme [5]. Early diagnosis significantly improves the outcome [3] and treatment is mainly dietary, consisting of avoidance of prolonged fasting and an emergency regimen during intercurrent illness [6]. Secondary free carnitine (C0) deficiency in blood may be corrected by carnitine supplementation in some patients, but evidence for this treatment is limited [7].

Since the introduction of the NBS programs, newborns are identified by the detection of increased concentrations of medium-chain length acylcarnitines and their ratios [8]. An elevated concentration of octanoylcarnitine (C8:0) is the most common biomarker for MCAD deficiency. Besides patients with classical *ACADM* genotypes, NBS identifies subjects with hyperoctanoylcarnitinaemia and variant *ACADM* genotypes, i.e. genotypes that have not been recognized before in clinically ascertained patients [9]. As population NBS programs aim to prevent development of a phenotype, physicians feel forced to institute treatment, independent of the genotype. It can be questioned, however, whether subjects with variant *ACADM* genotypes have the same clinical risks compared to patients with classical *ACADM* genotypes. Or even, whether they should be regarded patients at all. Different studies used laboratory parameters to estimate the significance of variant *ACADM* genotypes [10-14]. Clinical follow-up parameters are difficult to interpret, because early diagnosis and treatment influence the natural clinical course of subjects with variant *ACADM* genotypes.

It could be hypothesised that residual MCAD enzyme activity is a prognostic parameter in risk stratification of patients with variant *ACADM* genotypes. Therefore, we performed a cohort study integrating NBS test results, molecular studies, and clinical data with enzymatic data of all patients from the Dutch birth cohorts 2007–2010 identified in the population NBS program for MCAD deficiency. The results from subjects with variant *ACADM* genotypes were stratified, using data from patients with classical *ACADM* genotypes as a reference.

## Patients and methods

The Medical Ethical Committee of the University Medical Centre Groningen approved the study (METc 2011/133). Parents provided written informed consent.

### **Population NBS protocol**

In The Netherlands, the epidemiology and natural history of MCAD deficiency have been well characterised before introduction of the population NBS program in 2007 [2,4,15]. In our country, 99.75% of all newborns are screened between 72 and 168 h after birth ( http://www.rivm.nl). In dried blood spots the following parameters are determined: concentrations of C0, C8:0, and decanoylcarnitine (C10:0), and the C8:0/C10:0 ratio. For MCAD deficiency, the decision is based on the C8:0 concentration only as clinical and laboratory follow-up is initiated in newborns with C8:0 ≥ 0.50 μmol/l within 24 h.

### **Protocol for laboratory follow-up**

After an initial positive NBS test for MCAD deficiency, the newborns are referred to a metabolic centre. Laboratory follow-up includes a complete acylcarnitine profile in plasma and/or a dried blood spot by tandem mass-spectrometry, and urinary organic acid analysis using gas chromatography–mass spectrometry, as described by Derks et al. [9]. Determination of MCAD enzyme activity is performed in leukocytes or lymphocytes, with an HPLC-based assay using 3-phenylpropionyl-CoA (PP-CoA) as a substrate [9,16]. Residual MCAD enzyme activities are expressed as percentage from healthy controls. Analysis of the *ACADM* gene (OMIM 607008) is performed by sequencing all exons and adjacent intron regions. The nucleotide numbering starts from the first adenine of the ATG translation initiation codon of the *ACADM* cDNA sequence and amino acid numbering starts from the methionine encoded by this translation initiation codon. The abovementioned analyses are preceded by parental informed consent.

A ‘variant *ACADM* genotype’ was defined as an *ACADM* genotype that has not been recognized before in clinically ascertained patients in either The Netherlands [2] or in literature.

### **Cohort**

In this study, we included children from the Dutch birth cohorts 2007–2010 with clinical follow-up in a metabolic centre after population NBS for MCAD deficiency. For most children the diagnosis has been confirmed by MCAD enzyme and/or *ACADM* gene analysis. If these analyses were not performed, the confirmatory acylcarnitine profile was characterised by C8:0 concentrations >50 z-scores (i.e. ≥ 1.65 μmol/l) and a C8:0/C10:0 ratio ≥10 z-scores (i.e. ≥ 8.3) above the threshold for NBS, based on reference data from the National Institute for Public Health and the Environment.

Clinical and laboratory data from all patients were retrospectively retrieved from the Dutch Diagnosis Registration Metabolic Diseases database ( http://www.ddrmd.nl), and medical and laboratory files by one investigator (CT). Data from the patients in which the diagnosis had been confirmed enzymatically were included in the analysis of the prognostic value of MCAD enzyme analysis.

### **Data analysis**

Using data from the Dutch Central Bureau for Statistics (CBS) ( http://www.cbs.nl), the prevalence P was calculated by dividing the total number of patients by the total number of newborns N. For calculation of the 95% confidence interval (95% CI) the following formula was used:

$$95\%\text{ CI}=P\pm 1.96\;\times {\left(\left(P\times \left(1-P\right)\right)/N\right)}^{1/2}$$

Differences between normally distributed continuous data were analyzed using parametric tests. Data that were not normally distributed were analyzed using nonparametric tests. For dichotomous data, a chi-squared test was used. For analysis of correlations, Spearman’s rank test was used. The significance level was set at p < 0.05. Statistical analyses were performed using GraphPad Prism software (GraphPad Software Inc., version 5.00, 2007).

## Results

### **Cohort**

In the period 2007–2010, the diagnosis MCAD deficiency was confirmed in 84 patients, after 108 initial positive screening results for C8:0 ≥ 0.50 μmol/l. Data from the NBS test, molecular studies and clinical follow-up were compared to the residual MCAD enzyme activity, in order to determine the prognostic value. Results from patients with a classical *ACADM* genotype were used as a reference.

### **Relationship between*ACADM*genotypes and residual MCAD enzyme activities**

*ACADM* genotypes were available from 68 of the 84 patients (Table 1). Homozygosity for the common c.985A > G mutation was observed in 62% (42/68), the c.985A > G allele frequency was 77% (104/136). Variant *ACADM* genotypes were observed in 22% (15/68).

**Figure 1:**
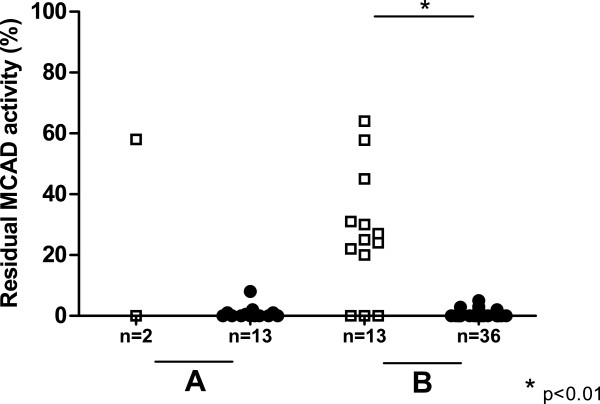
**Residual MCAD enzyme activities measured in leukocytes, and lymphocytes. **Legend: Residual MCAD enzyme activities measured in leukocytes (A), and lymphocytes (B) from true positives with variant *ACADM *genotypes (squares) and classical *ACADM *genotypes (dots).

Data on residual MCAD enzyme activity could be obtained from 64 of the 84 patients. Median residual MCAD enzyme activity in patients with classical *ACADM* genotypes was 0% (range 0-8% and 0-5% in leukocytes and lymphocytes, respectively). Subjects with variant *ACADM* genotypes displayed significantly higher MCAD residual enzyme activities (median 25%, range 0-63%, Mann–Whitney *U* test, p < 0.01) (Figure 1).

**Table 1:** ***ACADM *genotypes in 68 patients, identified upon population NBS for MCAD deficiency in The Netherlands**

**Genotype**		**Allele 1**			**Allele 2**			**MCAD enzyme activity (%)**		**References**
	**Number**	**Nucleotide change**	**Exon**	**Coding effect**	**Nucleotide change**	**Exon**	**Coding effect**	**A**	**B**	
**CLASSICAL**	42	c.985A > G	11	p.K329E	c.985A > G	11	p.K329E	0	0	[25-27]
	5	c.985A > G	11	p.K329E	c.233 T > C	4	p.I78T	2	2	[23]
	2	c.985A > G	11	p.K329E	c.789A > G	9	p.L263F	0	n.d.	[2]
	1	c.985A > G	11	p.K329E	c.799 G > A	9	p.G267R	8	n.d.	[28,29]
	2	c.233 T > C	4	p.I78T	c.233 T > C	4	p.I78T	<1	3	[23]
	1	c.233 T > C	4	p.I78T	c.789A > G	9	p.L263F	0	n.d.	[2,23]
**VARIANT**	2	c.985A > G	11	p.K329E	c.158 G > A	3	p.R53H	n.d.	23	This study
	3	c.985A > G	11	p.K329E	c.199 T > C	3	p.Y67H	58	31	[30]
	1	c.985A > G	11	p.K329E	c.216 + 1 G > T	4	Splice site variant	n.d.	0	This study
	2	c.985A > G	11	p.K329E	c.238A > G	4	p.R80G	n.d.	36	This study
	1	c.985A > G	11	p.K329E	c.470 C > T	7	p.A157V	n.d.	0	This study
	1	c.985A > G	11	p.K329E	c.493 G > A	7	p.A165T	n.d.	20	This study
	1	c.985A > G	11	p.K329E	c.600-18 G > A	8	p.XXX?	n.d.	63	[10]
	1	c.985A > G	11	p.K329E	c.928 G > A	10	p.G285R	0	n.d.	[23]
	1	c.233 T > C	4	p.I78T	c.1066A > T	11	p.I356F	n.d.	0	This study
	1	c.250 C > T	4	p.L84F	c.199 T > C	3	p.Y67H	n.d.	58	[30,31]
	1	c.799 G > A	9	p.G267R	c.865 G > A	10	p.V289I	n.d.	25	This study

A, measured in leukocytes; B, measured in lymphocytes; n.d., not determined. Median residual MCAD enzyme activities are depicted.

For further comparison of groups, patients were stratified based on residual MCAD enzyme activity. A threshold of 10% was considered practical and safe, because the highest residual MCAD enzyme activity determined in a patient with a classical *ACADM* genotype was 8%.

### **Relationship between population NBS test results and MCAD enzyme activities**

**Figure 2:**
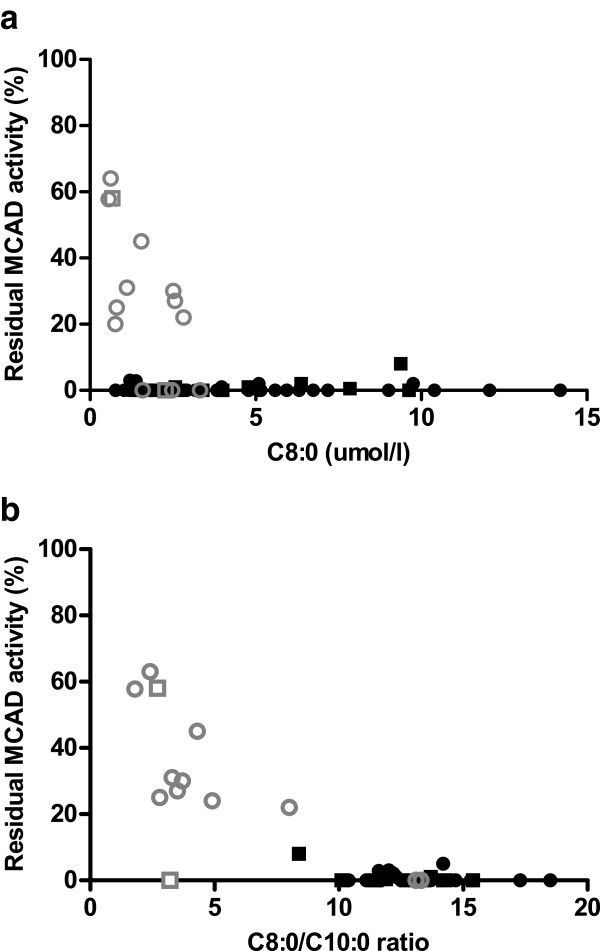
**Relationship between residual MCAD enzyme activity and C8:0 and C8:0/C10:0 ratio upon NBS. **Legend: Classical *ACADM *genotypes depicted in black, variant *ACADM *genotypes in grey. Squares: measured in leukocytes; Circles: measured in lymphocytes.

Figure 2a demonstrates that C8:0 concentrations were significantly higher in patients with residual MCAD enzyme activities <10% than in subjects with residual MCAD enzyme activities ≥10% (median 3.96, range 0.77–14.80 μmol/l vs. median 1.11, range 0.56– 2.82 μmol/l, respectively, Mann–Whitney *U* test, p < 0.01). Additionally, C8:0/C10:0 ratios were significantly higher in the group of patients with residual MCAD enzyme activities <10% (median 13.00, range 3.21–18.50), when compared to the group of subjects with residual MCAD enzyme activities ≥10% (median 3.41, range 1.81–8.03, Mann–Whitney *U* test, p < 0.01). A strong negative linear correlation was found between the NBS C8:0/C10:0 ratio and the residual MCAD enzyme activity measured in lymphocytes (Spearman r = −0.67, p < 0.001) (Figure 2b).

### **Relationship between clinical phenotypes and MCAD enzyme activities**

Seven newborns had a clinical presentation in the neonatal period before diagnosis (Table 2). Two of these newborns died before arrival in the hospital. Five had been admitted to the hospital before NBS test results became available, three of them with documented hypoglycaemia. All newborns with a neonatal presentation had a C8:0/C10:0 ratio ≥10 upon NBS and residual MCAD enzyme activities <1%. Six of these patients had a classical *ACADM* genotype. The seventh case had the variant c.985A>G/c.216+1G>T genotype, corresponding with 0% residual MCAD enzyme activity.

**Table 2:** Prevalence of clinical symptoms and interventions in patients, organised by residual MCAD enzyme activity

	**Residual MCAD enzyme activity**	
	**< 10%**	**≥ 10%**
Neonatal presentation (%)	13% (7/53)	0%
Hypoglycaemia (%)	8% (4/51)	0%
Carnitine supplementation (%)	51% (27/51)	0%
Patients with hospital admissions (%)	82% (42/51)	55% (6/11)*
Hospital admissions (n)	116	14*
Hospital admissions per life year	0.64/life year	0.55/life year

Data were processed as described in ‘Patients and Methods’. Hypoglycaemia was defined as blood glucose concentration <2.6 mmol/L or reported hypoglycaemia in medical charts. * p < 0.05.

After the diagnosis was established, no fatal manifestations of MCAD deficiency were reported. Patients with residual MCAD enzyme activities <10% were 1.2 times more frequently admitted to the hospital preventively, compared to subjects with residual MCAD activities ≥10% (Table 2). All patients with documented hypoglycaemia had residual MCAD enzyme activities <10%. During follow-up, no carnitine was prescribed for low plasma C0 concentrations in subjects with residual MCAD enzyme activities ≥10%. In contrast, 51% (27/51) of the patients with residual MCAD enzyme activities <10% received carnitine supplementation.

### **Epidemiology**

Between 2007–2010, 84 patients were identified from 76 families. The prevalence of MCAD deficiency was 1/8,750 (95%CI 1/7,210–1/11,130), which is considered to be a high estimate (Table 3). When subjects with residual MCAD activities ≥10% were excluded, the prevalence was 1/10,070 (95%CI 1/8,190-1/13,070).

**Table 3:** Epidemiology of MCAD deficiency in The Netherlands before and after the introduction of population NBS

**MCAD deficiency in The Netherlands**	**Frequencies (95% CI)**
*ACADM *mutation carrier frequency^a^	1/55 (1/46 – 1/68)
Expected prevalence^a^	1/12,100 (1/8,450 – 1/18,500)
Observed prevalence 1985-1999^b^	1/27,400 (1/23,000 – 1/33,900)
Screened newborns 2007-2010^c^	735,282
Patients 2007-2010^d^	84
Prevalence upon neonatal screening^d^	1/8,750 (1/7,210 – 1/11,130)

^a^ According to De Vries et al. [15]; ^b^ According to Derks et al. [4]; ^c^ According to CBS; ^d^ This study.

## Discussion

This is the first report in which residual MCAD enzyme activities in a large cohort of patients identified upon population NBS for MCAD deficiency have been used for risk stratification. Within the group of newborns with hyperoctanoylcarnitinaemia, a broad spectrum of *ACADM* genotypes has been identified. As the clinical significance of many variant *ACADM* genotypes is incompletely known, we integrated clinical, molecular and enzymatic data from a well-defined population. Residual MCAD enzyme activity correlated well with *ACADM* genotype and phenotype, and could therefore aid in the risk stratification of patients after positive NBS test.

In this study, residual MCAD enzyme activity was measured with PP-CoA, which is a very specific substrate to determine residual MCAD enzyme activity *in vitro*[16,17]. Traditionally, natural substrates such as hexanoyl-CoA and octanoyl-CoA were used to determine MCAD enzyme activity. However, even in patients who were homozygous for the classical c.985A > G *ACADM* mutation, high residual enzyme activities were found when using these substrates, possibly due to the overlap in substrate specificity with other acyl-CoA dehydrogenases [9]. Therefore, in our country these substrates have been replaced in the confirmatory enzymatic studies after positive NBS test results. However, the possible role of PP-CoA in the pathophysiology of MCAD deficiency is currently unknown and it is important to realise that the pathophysiology is far more complex than just deficient MCAD enzyme activity.

Before NBS test results became available, a neonatal presentation occurred in seven patients with residual MCAD enzyme activities <1%. Two of these patients presented with a fatal event. Despite the important benefits of population NBS programs for MCAD deficiency [3], the question remains justified, whether we detect *all* patients before clinical presentation and whether they are all *patients*. When compared to other NBS programs, blood sampling for the NBS test is relatively late in our country, i.e. between 72 and 168 hours of life. However, similar percentages of (fatal) neonatal presentations have been reported in studies from other countries, where the NBS test is performed earlier in the neonatal period [3]. Since several inherited metabolic diseases may present with a fatal neonatal presentation, escaping early detection by population NBS programs, we recommend dried blood spot analysis of acylcarnitines and amino acids in all newborns who die before the NBS test has been performed.

In a subset of the children with variant *ACADM* genotypes, residual MCAD enzyme activity is relatively high (Table 1). This raises the question whether these subjects are at risk of developing clinical symptoms as patients with classical genotypes, and should be considered “patients”. If the main arguments for a population NBS program are strictly considered [18], subjects with variant *ACADM* genotypes may not be regarded “true-positives”, because their genotypes have not been observed before in clinically ascertained patients. However, some of these mutations may have never been detected due to low carrier frequencies, but may give the same clinical risks as classical *ACADM* mutations [19]. Already before the introduction of NBS for MCAD deficiency, the term “patient” was a matter of debate. Reduced penetrance of the disorder is a well-recognised phenomenon, reflected by asymptomatic family members with the same classical *ACADM* genotype as clinically ascertained probands [4]. Moreover, it is recognised that single nucleotide polymorphisms (in combination with a classical *ACADM* mutation) may contribute to the development of hyperoctanoylcarnitinaemia by modulating mitochondrial fatty acid oxidation [19].

In this study, 15 subjects had a ‘variant *ACADM* genotype’. Three newborns (with c.985A>G/c.216+1G>T; c.985A>G/c.470C>T and c.233T>C/c.1066A>T genotypes) displayed C8:0/C10:0 ratios >10 and residual MCAD enzyme activity <1% (Figure 2b), as observed in patients with classical *ACADM* genotypes. In our opinion, they should be considered and treated like patients with classical *ACADM* genotypes. The patient with the c.985A>G/c.928G>A genotype displayed a C8:0/C10:0 ratio <10. Since MCAD enzyme activity was absent, he was considered “patient” like patients with classical *ACADM* genotypes. In the remaining 11 subjects with variant *ACADM* genotypes, residual MCAD enzyme activities ranged between 20%–63%. Should they receive (the same) treatment as patients with classical genotypes? In previous studies that aimed to determine risk stratification for subjects with variant *ACADM* genotypes, different sets of data have been used. Examples include correlation of metabolite concentrations to *ACADM* genotypes [10,11], or heterolo-gous overexpression studies to determine the effect of various *ACADM* mutations on residual MCAD enzyme activity and the thermal stability of the MCAD enzyme [12-14]. In most cases, however, the effect of one single *ACADM* mutation was studied, although MCAD deficiency is an enzyme deficiency caused by genetic alterations on both *ACADM* alleles.

In this study, classical *ACADM* genotypes were associated with residual MCAD enzyme activities <10%. Substantial octanoate oxidation [20] and normal ketone body metabo-lism [21] were demonstrated in previous stable isotope studies under normal fasted conditions in patients with classical *ACADM* genotypes. It is well recognised that clinically ascertained patients tolerated overnight fasting without problems before establishment of the diagnosis, as recently reviewed [22]. Similar observations were made in our country, since overnight fasting per se has never precipitated symptoms in patients [2,6]. As a subset of subjects with variant *ACADM* genotypes was associated with a milder biochemical and clinical phenotype, and relatively high residual MCAD enzyme activity, it could be argued that the general advice on avoidance of overnight fasting in subjects with residual MCAD enzyme activities ≥10% can be abandoned. However, the major argument in favour of avoiding prolonged fasting is early anticipation in circumstances of intercurrent illness with fever in young patients. Some (variant) *ACADM* mutations have shown to lead to temperature sensitive MCAD folding variants *in vitro*[14,23]. The clinical *in vivo* effects remain to be determined. Therefore, an emergency regimen and clinical follow-up remain basic principles of the treatment, regardless of *ACADM* genotype.

The prevalence of MCAD deficiency upon NBS in The Netherlands is threefold higher than found after clinical presentation (Table 3) [4], and in line with previous reports [24]. Prior to the introduction of the NBS program, the expected prevalence of MCAD deficiency was calculated to be 1/12,100 (95%CI: 1/8,450–1/18,500) in our country, based on the *ACADM* c.985A>G carrier frequency in the general population and the assumption of a 94% allele frequency for this common mutation in clinically ascertained cases [15]. The latter was confirmed in patients from the Dutch birth cohorts 1985–1999 [4]. The current study displays a more heterogeneous *ACADM* mutational spectrum with an observed allele frequency of the c.985A>G *ACADM* mutation of only 77%. Although the observed prevalence is comparable to the expected prevalence [15], it is important to realize that the availability of molecular tests for the complete *ACADM* gene has significantly increased since the 1990s. This might have biased identification of patients in our previous studies, hence, causing an underestimation of the expected prevalence. In addition, demographic alterations within the same geographic area might have contributed ( http://www.cbs.nl).

## Conclusions

Population-wide NBS programs identify newborns with hyperoctanoylcarnitinaemia and variant *ACADM* genotypes. These newborns are currently regarded patients, in whom follow-up and dietary treatment are initiated similarly to the group with classical *ACADM* genotypes. This study demonstrates the prognostic value of residual MCAD enzyme activity in newborns with hyperoctanoylcarnitinaemia and a variant *ACADM* genotype. Residual MCAD enzyme activities <10% were associated with clinical symptoms, regardless of *ACADM* genotype. Clinical symptoms or classical *ACADM* genotypes have thus far not been reported in subjects with residual MCAD enzyme activities ≥10%. For all positively screened newborns with any form of MCAD deficiency, basic principles of the treatment are parental instructions, cautious clinical follow-up, and application of an emergency regimen. In subjects with variant *ACADM* genotypes and ≥10% residual MCAD enzyme activities, the necessity to avoid overnight fasting is debatable.

## Abbreviations

*ACADM*: Gene encoding medium-chain acyl-CoA dehydrogenase; C0: Free carnitine; C8:0: Octanoylcarnitine; C10:0: Decanoylcarnitine; C8:0/C10:0: Ratio between octanoylcarnitine and decanoylcarnitine concentrations; MCAD: Medium-chain acyl-CoA dehydrogenase; NBS: Newborn bloodspot screening; PPA: Phenylpropionic acid; PP-CoA: Phenylpropionyl-CoA.

## Competing interests

The authors declare that they have no competing interests.

## Authors’ contributions

CML Touw participated in data collection, analysis and interpretation, generation of the figures, in writing of the manuscript, and approved of the final version. GPA Smit conceived the study, participated in writing the manuscript, and approved the final version. M de Vries, JBC de Klerk, AM Bosch, G Visser, MF Mulder, ME Rubio-Gozalbo, KE Niezen-Koning, B Elvers, RJA Wanders, and HR Waterham participated in data collection, and approved of the final version. DJ Reijngoud conceived the study, participated in writing the manuscript, and approved of the final version. TGJ Derks conceived the study, participated in analysis and interpretation of the data, in writing of the manuscript, and approved of the final version.
